# LSTM-DDPG for Trading with Variable Positions

**DOI:** 10.3390/s21196571

**Published:** 2021-09-30

**Authors:** Zhichao Jia, Qiang Gao, Xiaohong Peng

**Affiliations:** 1 School of Electronics and Information Engineering, Beihang University, Beijing 100191, China; jia313682644@buaa.edu.cn; 2 Hangzhou Innovation Institute, Beihang University, Hangzhou 310051, China; 3 Faculty of Computing, Engineering and the Built Environment, Birmingham City University, Birmingham B5 5JU, UK; xhpeng100@gmail.com

**Keywords:** trading strategy, deep reinforcement learning, variable positions, reward function

## Abstract

In recent years, machine learning for trading has been widely studied. The direction and size of position should be determined in trading decisions based on market conditions. However, there is no research so far that considers variable position sizes in models developed for trading purposes. In this paper, we propose a deep reinforcement learning model named LSTM-DDPG to make trading decisions with variable positions. Specifically, we consider the trading process as a Partially Observable Markov Decision Process, in which the long short-term memory (LSTM) network is used to extract market state features and the deep deterministic policy gradient (DDPG) framework is used to make trading decisions concerning the direction and variable size of position. We test the LSTM-DDPG model on IF300 (index futures of China stock market) data and the results show that LSTM-DDPG with variable positions performs better in terms of return and risk than models with fixed or few-level positions. In addition, the investment potential of the model can be better tapped by the reward function of the differential Sharpe ratio than that of profit reward function.

## 1. Introduction

Over the past decade, machine learning techniques have driven significant advances across many application areas which inspire investors and financial institutions to develop machine learning-aided investment strategies. More and more studies have applied machine learning to financial investment [1,2,3,4,5,6]. However, the highly non-stationary nature of financial markets hinders the application of typical data-hungry machine learning methods in financial investment.

Supervised learning is suitable for portfolio strategies because sufficient data can be prepared when investing in a portfolio. Imajo et al. [7] propose a system for constructing portfolios with a spectral decomposition-based method to hedge out common market factors and a distributional prediction method based on deep neural networks incorporating financial inductive biases. Nakagawa et al. [8] propose a principled stock return prediction framework called Ranked Information Coefficient Neural Network (RIC-NN) to alleviate the overfitting. The learning difficulties of initialization, the stopping of training models and the transfer among different markets have been addressed. Pun et al. [9] present a financial thought experiment with the aid of the Generative adversarial network (GAN) framework and adapt it to the portfolio risk minimization problem by adding a regression network to GAN. Tsang et al. [10] investigate a deep-learning solution to high-dimensional multiperiod portfolio optimization problems with bounding constraints on the control.

Reinforcement learning is suitable for trading strategies while supervised learning is suitable for portfolio strategies. The trading process can be considered as a multistep decision process and the reinforcement learning framework is suitable for solving trading problems [11,12,13,14,15]. In reinforcement learning-aided trading, an agent interacts with the market environment and simultaneously makes trading decisions without any supervised information [16,17,18,19,20,21,22].

Neuneier [23] uses Q-learning to make trading decisions of position direction, in which the Q-learning model optimizes the trading policy by sampling state-action pairs and returns while interacting with market conditions. Moody et al. [15] propose a Recurrent Reinforcement Learning (RRL) model to make trading decisions. The RRL model takes the previous action as part of the input to properly take into account the effects of transactions costs. Deng et al. [11] introduce a Recurrent Deep Neural Network (RDNN) for simultaneous environment sensing and recurrent decision making for online financial assert trading. The bulk of the RDNN is composed of two parts of recurrent neural network (RNN) for reinforcement learning. Liu et al. [24] propose imitative recurrent deterministic policy gradients to automatically develop trading strategies by an intelligent trading agent. In addition, imitation learning is introduced to create a balance between the exploration and exploitation of strategy.

The aforementioned studies mainly focus on making decisions of position direction. A fixed position size or the maximum position size is assumed explicitly or implicitly in these studies. Both the position direction and position size should be determined based on the market condition during trading. To achieve high profit with low risk, the position size should vary with market conditions. When the market has a large and steady upward or downward trend and the market condition is easy to identify, we should set a large position size to obtain high profits. When the market is volatile, a small position size or even zero size is preferred to reduce trading risk.

Recently, some scholars considered changeable position sizes during trading. Li et al. [25] proposed a position-controlled action space. The action space is extended to {−3, −2, −1, 0, 1, 2, 3}, which represents the position held in the next state. Jeong et al. [26] predict the position size by adding a DNN regressor to a deep Q-network. Besides, the position size is limited by a maximum position size of 10. These studies only consider a few levels or limited levels of position size, and there is no work considering variable position sizes as far as we know. If the position size can change arbitrarily with subtle variations of market condition, the efficiency of investment capital can be improved and the trading risk can be reduced further.

In this paper, we propose an LSTM-DDPG model to make trading decisions with variable positions. To be specific, we describe the trading process as a Partially Observable Markov Decision Process (POMDP) with the acknowledgement that the financial market environment is not completely observable. The LSTM-DDPG model is composed of a long short-term memory (LSTM) network and deep deterministic policy gradient (DDPG) framework. The LSTM is used to extract environmental state features from environmental observations and the DDPG is used to make trading decisions. The DDPG consists of a critic network and an actor network. The critic network estimates the action-value function and the actor network adjusts the deterministic policy which outputs a continuous action at each step. The action is a real number in the range of [−1, +1]. The sign of the action (+, −) represents the position direction, and the absolute value represents the position size with variable amounts. In addition, we consider two different reward functions: DSR and profit. The experimental results of our model on IF300 (index futures of the Chinese stock market) data show that the model can achieve well-balanced trading performance between profit and risk factors.

The remaining parts of this paper are organized as follows. Section 2 describes the details of the proposed LSTM-DDPG model. Section 3 is the experimental part where we evaluate the performance of our model. Section 4 concludes this paper.

## 2. Methodology

We look the trading process as a POMDP, and propose the LSTM-DDPG model to solve it.

### 2.1. Partially Observable Markov Decision Process

In a financial market, the security price is influenced by macroeconomic policies and microeconomic activities, which contain the information on unpredictable events and trading behaviors of all the market participants. Therefore, it is difficult to model the true financial market from the perspective of an investor or a trading agent and the trading process can be viewed as a Partially Observable Markov Decision Process (POMDP). 

A POMDP is a tuple S,A,T,R,Ω,O,γ.Here S is a set of states, and $s_{t}\in S$ is the state at time step t.$\;s_{t}$ is not well known by the trading agent. A is a set of actions, and $a_{t}\in A$ represents the action at step t. We define the trading action as a continuous variable within a range of [−1, +1], which represents the variable positions. The direction of variable positions is represented by the sign of the action, i.e., ‘+’ means long positions, ‘−’ means short positions and ‘0’ means no holding. The size of variable positions is represented by the absolute value of the action and is measured as a percentage of the amount of total capital. For example, $a_{t}=+0.6\;$means we have a long position with the 60% total capital invested in the financial market. T is a state transition matrix, which consists of conditional transition probabilities between states. R is the reward function. $R_{t}=R\left(s_{t},a_{t},s_{t+1}^{\prime }\right)$ represents the instantaneous reward at step t after executing the action $a_{t}$. Ω is a set of observations and O is a set of conditional observation probabilities. In our study the closing prices of the past 20 trading days are inputted into a LSMT network as the observation, and the output $h_{t}$ is the modelling of market state. γ is the discount factor ranging from 0 to 1, which is used to calculate the future discount rewards.

Optimizing investment is essentially a multi-objective optimization problem that requires maximizing profits and minimizing risks. The profit and DSR are taken as reward functions separately in our trading model. The profit reward function considers only profit, while the reward function of DSR takes both profit and risk into account.

Considering the transaction fees and slippage, the profit $r_{t}$ is defined as

(1) $$r_{t}=\frac{K}{p_{t}}\left\{\left(p_{t+1}-p_{t}\right)a_{t}-\delta p_{t}\left|a_{t}-a_{t-1}\right|\right\}.$$

where K represents the total capital. $p_{t}$ and $a_{t}$ are the price and the trading action at time t. δ is a parameter which accounts for transaction fees and slippage. When the reward function is profit, the expected future discount rewards are cumulative return.

When the reward function is DSR, the expected future discount rewards is related to the Sharpe ratio. The Sharpe ratio $S_{t}$ is defined as

(2) $$S_{t}=\frac{E\left[r_{i}\right]}{\sigma \left[r_{i}\right]},i\leq t$$

where $E\left[r_{i}\right]$ is the mean of profits and $\sigma \left[r_{i}\right]$ is the standard deviation of profits which represents the volatility of profits and the trading risk (see [15]). 

Expanding the Sharp ratio to the Taylor series in the adaptation rate η, we have

(3) $$S_{t}\approx S_{t-1}+\eta \frac{dS_{t}}{d\eta }{|}_{\eta =0}+o\left(\eta^{2}\right).$$

Noting that only the first-order term in this expansion depends upon the return $r_{t}$ at time t, so the DSR $d_{t}$ (see [15]) can be defined as

(4) $$d_{t}\equiv \frac{dS_{t}}{d\eta }=\frac{B_{t-1}\Delta A_{t}-\frac{1}{2}A_{t-1}\Delta B_{t}}{{\left(B_{t-1}-A_{t-1}^{2}\right)}^{\frac{3}{2}}}.$$

In this expression,$\;A_{t}$ and $B_{t}$ are the estimations of exponential moving average for the first and second moments of $r_{t}$, $\Delta A_{t}$ and $\Delta B_{t}$ are their update quantities. They can be written as

(5) $$A_{t}=A_{t-1}+\eta \Delta A_{t}=A_{t-1}+\eta \left(r_{t}-A_{t-1}\right),$$

(6) $$B_{t}=B_{t-1}+\eta \Delta B_{t}=B_{t-1}+\eta \left(r_{t}^{2}-B_{t-1}\right),$$

(7) $$\Delta A_{t}=r_{t}-A_{t-1},$$

(8) $$\Delta B_{t}=r_{t}^{2}-B_{t-1}.$$

### 2.2. LSTM-DDPG

**Figure 1:**
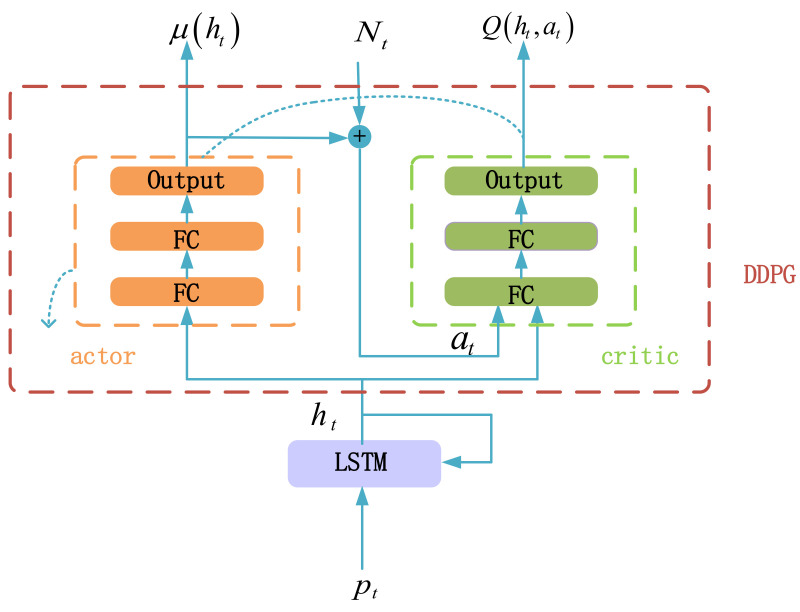
LSTM-DDPG model.

We propose the LSTM-DDPG model to solve the POMDP for trading, which is shown in Figure 1. The LSTM-DDPG is composed of the LSTM network and the DDPG framework. The LSTM network is used to extract the market features and the DDPG framework is used to make trading decisions.

#### 2.2.1. LSTM

LSTM is a special RNN that can learn the long-term dependency within the input data. The past closing prices of T=20 trading days are looked as the observations of financial market and are input into LSTM, and the output of LSTM represents the market state, hence the LSTM is unrolled for T time steps. LSTM can be formulated as follows

(9) $$f_{t}=\Lambda \left(W_{f}\cdot \left[h_{t-1},p_{t}\right]+b_{f}\right),$$

(10) $$i_{t}=\Lambda \left(W_{i}\cdot \left[h_{t-1},p_{t}\right]+b_{i}\right),$$

(11) $${\tilde{c}}_{t}=\tan h\left(W_{c}\cdot \left[h_{t-1},p_{t}\right]+b_{c}\right),$$

(12) $$c_{t}=f_{t}*c_{t-1}+i_{t}*{\tilde{c}}_{t},$$

(13) $$o_{t}=\Lambda \left(W_{o}\cdot \left[h_{t-1},p_{t}\right]+b_{o}\right),$$

(14) $$h_{t}=o_{t}*\tan h\left(c_{t}\right),$$

where $p_{t}$ is the closing price of market at time t. $h_{t}$ is the output vector, $f_{t}$ is the forget gate, $i_{t}$ is the input gate and $o_{t}$ is the output gate, $c_{t}$ is the cell state, ${\tilde{c}}_{t}$ is the update value of cell state, $W_{*}$ are weight matrices, $b_{*}$ are bias vectors. $\Lambda \left(x\right)$ is the sigmoid function and defined as $\Lambda \left(x\right)=\frac{1}{1+e^{-x}}$, which guarantees that the values of the gates are in the range of 0 to 1. The input gate $i_{t}$ gives the information that needs to be stored in the cell state $c_{t}$ and the forget gate $f_{t}$ controls the information which needs to be forgot from the last cell state $c_{t-1}$ to the current cell state $c_{t}$. The output gate $o_{t}$ is used to generate the output vector $h_{t}$ from the cell state $c_{t}$. Through the control of forget gate, input gate and output gate in the network, features are extracted according to the timing relationship of the input data.

#### 2.2.2. DDPG 

The DDPG framework is used to make trading decisions based on the market features captured by LSTM. The DDPG framework includes both the critic network and the actor network, and both are composed of two fully connected layers (FC) and one output layer, as shown in Figure 1. The critic network fits the action-value function. The actor network adjusts the trading policy by ascending the gradient of the action-value function.

The DDPG framework stores transitions $\left(o_{t},a_{t},R_{t},o_{t+1}\right)$ in the prioritized replay buffer during model training and then extracts transitions from the buffer to update the model parameters. To improve the exploration efficiency, Gaussian noise $N_{t}$ is added to the output of actor network $\mu \left(h_{t}\right)$ to construct the action

(15) $$a_{t}=\mu \left(h_{t}\right)+N_{t}.$$

The probability $V_{t}$ of a transition being sampled in the prioritized replay buffer is related to its priority $v_{t}$.

(16) $$V_{t}=\frac{v_{t}}{\sum_{i=1}^{t}v_{i}}$$

The transition priority is defined as follows

(17) $$v_{t}=\left|y_{t}-Q\left(h_{t},a_{t}|\theta^{Q}\right)\right|+\varepsilon ,$$

where $\theta^{Q}$ is the vector of parameters of the critic network, ε is a small positive constant to prevent $v_{t}$ from being zero. Q is the action-value function. $y_{t}$ is the estimation of cumulative return, which can be calculated as follows

(18) $$y_{t}=R_{t}+\gamma Q^{\prime }\left(h_{t+1},\mu^{\prime }\left(h_{t+1}|\theta^{\mu^{\prime }}\right)|\theta^{Q^{\prime }}\right),$$

where $\theta^{\mu }$ is the vector of parameters of the actor network. The vectors of parameters of the actor target network and the critic target network $\theta^{\mu^{\prime }}$ and $\theta^{Q^{\prime }}$ are recursively updated as follows

(19) $$\theta^{\mu^{\prime }}=\tau \theta^{\mu }+\left(1-\tau \right)\theta^{\mu^{\prime }},$$

(20) $$\theta^{Q^{\prime }}=\tau \theta^{Q}+\left(1-\tau \right)\theta^{Q^{\prime }}.$$

where τ is the renewal factor which affects the update rate of target networks.

The loss function of critic network is

(21) $$L=E\left[w_{t}*{\left(y_{t}-Q\left(h_{t},a_{t}|\theta^{Q}\right)\right)}^{2}\right],$$

where $w_{t}$ is the importance sampling weights of a transition, which is defined as

(22) $$w_{t}={\left(N*V_{t}\right)}^{-\beta },$$

where N is the batch size and β is a constant. 

The actor network updates its parameters in the direction of the action-value gradient. The gradient $\nabla_{\theta_{\mu }}J$ is defined as follows

(23) $$\nabla_{\theta_{\mu }}J=E\left[\nabla_{a}Q\left(h,a|\theta^{Q}\right){|}_{h=h_{i},a=\mu \left(h_{i}\right)}\nabla_{\theta_{\mu }}\mu \left(h|\theta^{\mu }\right){|}_{h=h_{i}}\right].$$

### 2.3. Training Process of LSTM-DDPG 

The detailed process to train the LSTM-DDPG model is described in Algorithm 1. The LSTM network and DDPG framework in the model are trained jointly. The parameters of the LSTM network are updated according to the loss passed back from the critic network. In the training process, when the total return of the training set for the latest epochs tends to be stable, the model is considered to be converged and the training is stopped. The set of parameters obtained during training will be used in the test data by the model for trading.
**Algorithm 1** Training Process of LSTM-DDPG**Input:** Closing prices $p_{1},\ldots ,p_{t},\ldots ,p_{M}$
**Initialization:**  Initialize the parameters of LSTM network $\theta^{L}$;       Initialize the parameters of actor network and actor target network $\theta^{\mu }$,$\;\theta^{\mu^{\prime }}$;       Initialize the parameters of critic network and critic target network $\theta^{Q},\theta^{Q^{\prime }}$;       Initialize the batch size N, size of prioritized replay buffer, discount        factor γ, renewal factor of target networks τ, parameter for importance       sampling weights β, learning rate of actor network, learning rate of        critic network, parameter accounting for transaction fees and slippage δ;       Initialize the prioritized replay buffer;       epoch = 0;1 **repeat:**2    **for** t=1…M
**do** 3      Update $o_{t}$;4      Output the feature of market $h_{t}$ by the LSTM;5      Output the trading action $\mu \left(h_{t}\right)$ by the actor network according to $h_{t}$;6      Add Gaussian noise to $\mu \left(h_{t}\right)$ to construct the action $a_{t}$; 7      Update $o_{t+1}$ and calculate the profit $R_{t}$;8      Store transition $\left(o_{t},a_{t},R_{t},o_{t+1}\right)\;$ in the prioritized replay buffer;9      Sample a minibatch of transitions from the prioritized replay buffer;10      Update $\theta^{Q},\theta^{L}$ according to Equation (21) based on Adam;11      Update $\theta^{\mu }$ according to Equation (23) based on Adam;12      Update $\theta^{\mu^{\prime }}$,$\;\theta^{Q^{\prime }}$ according to Equations (19) and (20);13    **end** 14     Calculate the total return of training set; 15     epoch = epoch + 1;16 **until** convergence.

## 3. Experiments

We conducted experiments to test our model. In this section, the experimental setup is represented. Then the performance of the reward functions of DSR and profit in LSTM-DDPG are compared. We also compare the performance of LSTM-DDPG when the fixed, few-levels, and variable position sizes are employed.

The direction and size of position should be determined in trading decisions based on the market conditions. The trading action $a_{t}$ takes the value from [−1, +1]. The sign of $a_{t}$ represents the position direction and the absolute value represents the position size. For the trading of fixed position sizes $a_{t}$ can only take values {−1, 0, +1}, i.e., there are three actions in the trading: investing all the capital in a long position, investing all the capital in a short position, holding no position. This is the case studied by most researches. For the trading of few-levels position sizes $a_{t}$ can take values {−1, −0.5, 0, +0.5, +1}, i.e., the trading system can take two more actions, investing half the capital in long or short positions. This is similar to the studies in [25,26]. For the trading of variable position sizes in the proposed LSTM-DDPG model, the position size changes continuously with the variation of market condition and $a_{t}$ is allowed to take any real value between −1 and +1.

### 3.1. Experimental Setup

**Figure 2:**
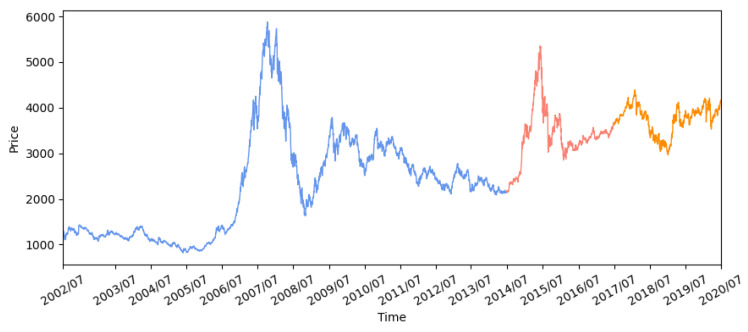
Daily closing price of IF300.

The proposed LSTM-DDPG trading model was tested on the China IF300, which is calculated based on the prices of the top 300 stocks from both the Shanghai and Shenzhen exchange centers. We use daily closing prices over 18 years spanning July 2002 to June 2020, which are shown in Figure 2. The data set is divided into a training set from July 2002 to June 2014, a validation set from July 2014 to June 2017, and a test set from July 2017 to June 2020.

The output of LSTM-DDPG is the trade action and the inputs are the closing prices of the previous 20 trading days. In LSTM-DDPG, the node number of the LSTM layer is set to 64. Both the actor network and the critic network have one output layer and two hidden layers, with 64 and 32 hidden nodes, respectively. 

The other hyperparameters used in the experiments are summarized in Table 1. The optimal batch size of 128 is set to balance the gradient oscillation and falling into a local minimum. The values of the prioritized replay buffer size, the discount factor, the renewal factor of target networks and the parameter for importance sampling weights are chosen by pre-training.

The learning rates for the actor network and the critic network are set according to [21]. The Adam optimizer is used for training. The transaction fees set by the futures exchange is 0.0023%, The parameter accounting for transaction fees and slippage is set to 0.01% in the experiments.

The proposed model is built and run on TensorFlow 2.3.1, a machine learning platform. The programming language is python 3.6.12. The LSTM-DDPG model is trained and evaluated on a server with two Intel Xeon Gold 6226R CPUs, two NVIDIA RTX 2080 Ti GPUs and 128 GB RAM.

Evaluation metrics in this study are total return rate, Sharpe ratio and maximum drawdown. The risk-return tradeoff is the trading principle, which states that the potential return rises with an increase in risk. Thus, a trading method can be assessed from the perspectives of profit and risk. The total return rate focuses on profit. The maximum drawdown emphases risk. The Sharpe ratio characterizes how well the return of a trading method compensates the investor for the risk taken.

Total return rate (TR) is the ratio of the return during the trading period, which can be formulated as

(24) $$TR=\frac{\sum_{t}r_{t}}{K},$$

where K represents the initial capital and $r_{t}$ is the profit during the tth sampling interval of the trading process.

The Sharpe ratio (SR) considers both profit and risk, which reflects the profitability under the unit trading risk and is defined as Equation (2). 

The maximum drawdown (MDD) describes the worst case in the process of trading, which reflects the trading risk and is generally related to the volatility of profits, i.e., the standard deviation of return $\sigma \left[r\right]$. The MDD is calculated as follows

(25) $$MDD=MAX\left(\frac{K_{t_{i}}-K_{t_{j}}}{K_{t_{i}}}\right),t_{i}<t_{j},$$

where $K_{t_{i}}$ is the capital at time$\;t_{i}$.

### 3.2. Experimental Results

#### 3.2.1. Profit vs. DSR as Reward Function in LSTM-DDPG

**Figure 3:**
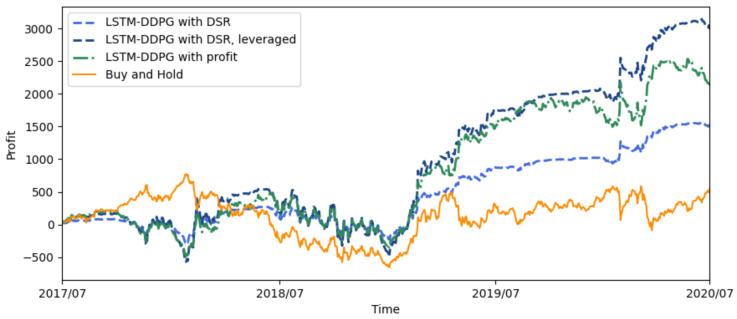
The profit curves of LSTM-DDPG with different reward functions.

We conducted experiments to compare the performance of the LSTM-DDPG with variable positions, in which the profit and DSR are taken as reward functions separately. Figure 3 illustrates the profit curves of Buy and Hold, the LSTM-DDPG with the reward functions of DSR, and profit for the test period from 2017 to 2020. Specifically, buy and hold refers to the trading method whereby we take a long position at the beginning and hold the position until the end of the test period. The profit curve of buy and hold also represents the IF300 itself.

As shown in Figure 3, the profit curves of LSTM-DDPG for both DSR and profit as reward functions are significantly higher than that of Buy and Hold most of the time, which means that the LSTM-DDPG is effective and can make a profit sustainably. Note that the price moves in a normal way from July 2017 to June 2020 while there is a “high peak” from July 2002 to June 2014. The LSTM-DDPG performs well in the test set although the market behavior in the test set is quite different from that in the training set since the agent in the reinforcement learning system can refine its responses and predictions and adapt to new environments by exploration. For the LSTM-DDPG, the profit curve of profit reward function is higher than that of DSR reward function in general, however the latter is much smoother than the former. It seems to be that the profit reward function can achieve better profit performance while the DSR reward function can achieve better risk performance in the proposed LSTM-DDPG model. 

Table 2 shows the performance results of buy and hold, and the LSTM-DDPG with different reward functions quantificationally. For the LSTM-DDPG, the profit reward function has the higher total return rate (42.5%) and larger maximum drawdown (15.3%) than the DSR reward function without leverage (29.8% and 9.5%). The Sharpe ratio is an indicator that considers both profit and trading risk. It can be seen from Table 2 that the DSR reward function has a higher Sharpe ratio (0.328) than the profit reward function (0.248). This means that the DSR reward function achieves better overall performance considering both profit and trading risk compared with the profit reward function.

Leverage is an investment mechanism using borrowed money to increase buying power in a margin account. The result is to multiply the potential returns and the potential downside risk will be multiplied at the same time. In China’s futures market you can borrow up to 90% of the purchase price of a security. You don’t have to margin all the way up to 90%. You can borrow less, say 50%. If you use $5000 cash in your margin account to purchase $10,000 worth of securities you would have a 2× leverage. IF300 is a financial instrument trading on leverage. To fairly compare the profitability of two reward functions in LSTM-DDPG, the profit curve of the DSR reward function is adjusted, keeping the volatility of profits the same as the profit reward function through leverage, which is shown in Figure 3.

It can be seen that the leveraged profit curve of the DSR reward function is significantly higher than that of the profit reward function most of the time, which means that in the LSTM-DDPG the DSR reward function can obtain higher profits than the profit reward function under the same trading risk. As shown in Table 2, the total return rate of the DSR reward function with leverage is 59.8%, which is higher than that of the profit reward function (29.8%), as expected. The DSR reward function can tap the investment potential of LSTM-DDPG with variable positions and can achieve well-balanced trading performance between profit and risk factors.

#### 3.2.2. Comparisons among Fixed, Few-Levels and Variable Position Sizes

When the position size changes arbitrarily with market condition in the process of trading, the efficiency of investment capital can be improved and the trading risk can be reduced. Table 3 shows the performance of buy and hold and the LSTM-DDPG with fixed, few-levels, and variable position sizes for the test period, in which DSR is taken as the reward function. When the variable position mechanism is employed in the LSTM-DDPG, a variable position size in the range from 0 to 1 will be determined according to market conditions. The few-levels position mechanism says that the position size can only be chosen from several values and here three values: 0, 0.5 and 1, are set. In the fixed position mechanism, no position or maximum position is used during trading.

It can be seen from Table 3 that in the LSTM-DDPG model the variable position mechanism has a higher Sharpe ratio (0.328) than the few-levels position mechanism (0.265), and that the few-levels position mechanism has a higher Sharpe ratio than the fixed position mechanism (0.215). The LSTM-DDPG, with variable position sizes, achieves the smallest maximum drawdown (9.5%) when there is no leverage in the trading process. We can also see from Table 3 that the LSTM-DDPG with variable position sizes can obtain the highest total return rate (46.3%) when leverage is used. These demonstrate that the variable position mechanism can achieve better overall performance considering both profit and trading risk compared with the fixed and few-levels position mechanisms.

**Figure 4:**
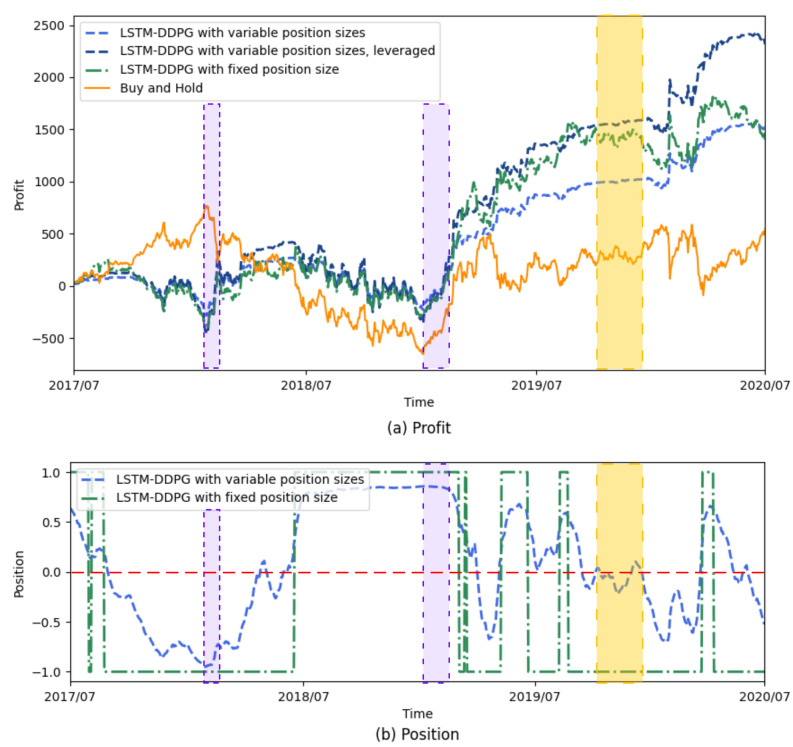
The profit curves and the position curves of the LSTM-DDPG with fixed and variable position sizes.

Figure 4 illustrates the profit curves and position curves of the LSTM-DDPG with fixed and variable position sizes, in which the DSR is taken as a reward function. We can see from the purple shadowed areas in the figure that the LSTM-DDPG with variable position sizes prefers a much larger position size in order to make as much money as possible when the market (as represented by the profit curve of buy and hold) presents a large upward or downward trend and the market direction is easily judged. We can also see from the yellow shadowed areas that the LSTM-DDPG with variable position sizes adjusts its position size to near zero to avoid trading risk when the market is volatile and it is difficult to determine the market direction or when there is no definite direction in the market. In this way the variable position mechanism in the LSTM-DDPG can adjust the position size according to the market conditions and thus can achieve high profit with low risk in the process of trading. Therefore, a good performance in term of the Sharpe ratio is expected for the LSTM-DDPG with variable position sizes.

## 4. Conclusions

In this paper, we have considered the trading process as a POMDP and proposed the LSTM-DDPG model to make trading decisions regarding the direction and variable size of position. The different reward functions of DST and profit have been considered as reward functions in the LSTM-DDPG. Our model has been trained and tested on China IF300 data. The experimental results show that the LSTM-DDPG model can achieve good trading performance with well-balanced profit and risk. The variable positions mechanism in LSTM-DDPG can adjust the position size according to the market conditions to try to increase the trading profitability and avoid trading risk. The LSTM-DDPG with variable positions and the DST reward function can achieve a higher total return rate for the testing period than other trading methods when leverage is used.

There are some investigations that can be pursued in the future. First, the high price, low price, trading volume etc. besides the closing price can be taken as the input of our model. Second, the trading action at the previous time step can affect the transaction fees of the current action. A better trading decision may be made if the previous action is considered. Therefore, the action at the previous step will be taken as the input of the actor network in our model in our future study. Moreover, to further demonstrate the effectiveness of our model, we will extend the experiments to other markets such as stocks, commodity futures, foreign exchange futures, etc.

## Figures and Tables

**Table 1:** Hyperparameters of LSTM-DDPG.

Hyperparameter	Value
batch size *N*	128
size of prioritized replay buffer	$1.0\times 10^{5}$
discount factor γ	0.8
renewal factor of target networks τ	0.01
parameter for importance sampling weights β	0.8
learning rate of actor network	$1.0\times 10^{-4}$
learning rate of critic network	$1.0\times 10^{-3}$
parameter accounting for transaction fees and slippage δ	0.01%

**Table 2:** Performance of buy and hold, LSTM-DDPG with different reward functions.

Method	Reward Function		Total Return Rate (TR)	Sharpe Ratio (SR)	Maximum Drawdown (MDD)
Buy and Hold	\		10.9%	0.040	24.7%
LSTM-DDPG	profit		42.5%	0.248	15.3%
	DSR	without leverage	29.8%	0.328	9.5%
		with leverage	59.8%	0.328	18.1%

**Table 3:** Performance of buy and hold, LSTM-DDPG with fixed, few-levels and variable position sizes.

Method	Position Size		Total Return Rate (TR)	Sharpe Ratio (SR)	Maximum Drawdown (MDD)
Buy and Hold	\		10.9%	0.040	24.7%
LSTM-DDPG	fixed		28.0%	0.215	12.9%
	few-levels		33.2%	0.265	12.4%
	variable	without leverage	29.8%	0.328	9.5%
		with leverage	46.3%	0.328	14.3%

## Data Availability

Not applicable.
